# Early exposure to geriatric care: developing an undergraduate internship in ethics and geriatric practice

**DOI:** 10.5116/ijme.52c6.da5e

**Published:** 2014-01-26

**Authors:** Erica K. Salter, Maura Waldron, Miguel A. Paniagua

**Affiliations:** 1 Gnaegi Center for Health Care Ethics, Saint Louis University, USA; 2 College of Nursing, Rush University, USA; 3 Department of Internal Medicine, Saint Louis University School of Medicine, USA

## Introduction

According to the United States Census Bureau, there was a 15.1% increase in the sixty-five and older population between 2000 and 2010^1^ and is expected to climb to 16.3% by 2020.^2^ In addition, older adults have shown a greater need for health care resource utilization in America; while only 12.8% of the U.S. population in 2008, older adults accounted for 26% of office visits, 35% of hospital stays, and 38% of emergency medical services.^3^The increase in care necessary for the aging population paired with the ebb of resident interest in geriatrics^4^ has prompted many to call for a more focused effort toward expanding awareness of and recruitment to geriatrics.^5^^-^^8^ Toward these ends, Saint Louis University (SLU) developed a university-level undergraduate course in ethics and geriatric care designed to expose students to the field prior to beginning formal post-baccalaureate medical education. In addition to regular classroom discussion of issues in geriatric care, students spend significant time learning through direct exposure to geriatrics by spending one hour shadowing clinicians and two hours visiting an elder at the nursing home. Students are assessed on classroom participation, completion of required hours, regular reflection papers integrating nursing-home experiences with classroom discussion, and completion of a quality improvement (QI) project.This course is unique for three reasons. First, it is one of the only university-level undergraduate courses engaging students in direct experiences with long term care residents. Second, it was designed in collaboration with undergraduate pre-professional students, who were surveyed to incorporate their needs into the course curriculum (pre-development needs analysis). Finally, it integrates reflection on and discussion of ethical issues related to geriatric long-term care with the students’ time at the nursing home, thereby grounding the practical experiences in theory and animating theory with real-world examples.

### Course design

The course was designed to meet several stakeholders’ interest simultaneously. First, the Division of Geriatric Medicine at SLU School of Medicine expressed a desire to increase awareness of common geriatric care issues and to pursue more volunteers to spend meaningful time with nursing home residents. Second, the pre-professional students at SLU completed a needs-based assessment to delineate student-driven course objects. The survey answers were qualitatively analyzed for common themes: 1) gaining experience with patients, 2) improving medical school applications, 3) increasing shadowing opportunities, 4) understanding the geriatric population, and 5) exposure to medical-ethical discussion. Finally, the University’s Center for Health Care Ethics was seeking opportunities for students to engage in real-world health care experiences while receiving course credit toward a minor degree in Health Care Ethics.The interests of these three stakeholders were combined to develop an internship-based service-learning course with resident companion contact hours, shadowing hours, classroom discussion, and ethics education. Students gain relevant knowledge and skills through real-world learning contexts and insight through volunteer and community service experiences. Upon completion of the course, students should be able to identify: (1) medical and professional challenges to geriatric care, (2) related ethical issues inherent in geriatric and end-of-life care, (3) effective communication and patient-centered skills with elder patients, and (4) principles of ethical care-giving.

### Course description and components

The course is offered for eighteen weeks every fall and spring semester, with a course size of twenty predominantly sophomore to senior pre-medical students, many with a health care ethics minor.

### Volunteer and shadowing component

Students must spend three hours per week in the nursing home. One hour is spent shadowing healthcare providers in the nursing home, primarily physicians, physical therapists and social workers. The central aim is to expose students to the clinical realities of patient care and interdisciplinary communication. The remaining two hours are resident companion hours, where students spend time with an assigned facility resident. This time is spent accompanying residents to therapy appointments, leisure activities, and mealtimes. Companion hours align directly with the service-learning objectives of the university. This objective is for students to learn what it is like to be an elder in a nursing home through regular interaction and relationship building. As a Jesuit institution, SLU has a commitment to providing service-learning opportunities through regular course work where students meet curricular objectives and participate in service to the community.In addition to regular shadowing and companion hours, students are required to complete a QI project at the nursing home. In small groups, students develop and execute an organized nursing home activity focused on offering new opportunities for socialization and recreation. The QI project introduces students to practical considerations of nursing home care and encourages improvements at a systematic level. Students must gain approval from the course instructor and the activities director at the nursing home through a proposal of the supplies needed, the possible risks, and projected outcomes. After the project is completed, students formally report on the benefits and drawbacks of their project.

### Classroom component

Twice a week students meet for one hour classroom sessions. A guest speaker presents on an issue related to geriatric care for the first classroom session. Guest speakers come from disciplines intersecting with geriatrics including physical therapy, occupational therapy, social work, law, pastoral care, hospice, ethics, case management, and medicine. The purpose of the guest speaker portion of the course is for students to learn the roles of the different professionals involved in geriatric care.

The second weekly hour is spent in reflective discussion. Seminar discussion proceeds from the students’ experiences at the nursing home and offers an opportunity to reflect on issues such as death and dying, practical challenges to long term care, and suffering and hope. To augment discussion, students are assigned short readings on a variety of subjects including the dilemmas of an aging society,^9^ the experience of Alzheimer’s disease,^10^ the physician experience of death,^11^ and the ethics of caregiving.^12^

The seminar component, also, integrates case discussion. Students examine a case with geriatric ethical dimensions (e.g. advance directives of Alzheimer’s patients, withholding futile care, family disagreements of care) from multiple points of view. In addition, students write five reflection papers throughout the semester integrating their nursing home experiences with course material. Reflection paper assignments include: describing an “ethical dilemma” experienced in the nursing home and constructing brief “illness narratives.”^13^

The classroom component provides course synthesis and offers students a supportive venue to discuss their experiences. This component is planned and facilitated by the primary course instructor, a faculty member in the Health Care Ethics Department.

## Discussion

At the end of the semester, students complete a post course essay series exploring topics discussed throughout the semester including communication and the role of independence. The post-course essay series was qualitatively analyzed for common themes which were combined with discussion notes from the course to find emergent topics.

One of the most substantial themes was the impact of the students’ clinical experiences on their learning. Classroom discussion was discernibly enhanced by the students’ ability to connect theoretical concepts to their time in the nursing home. For instance, students directly observe the importance of Activities of Daily Living on the quality their companion’s life which is also discussed in the classroom. Experiences in the nursing home were often brought up during classroom discussion to illustrate important concepts, as a springboard for further examination, and as an opportunity to offer support. Students directly experienced both positive and negative aspects of geriatric practice from the perspective of the clinician and the patient.

Students demonstrated an increased awareness of the unique challenges of geriatric medicine. Students displayed an ability to recognize and apply important concepts pertinent to geriatrics, including frailty, learned helplessness, and advanced directives. They articulated an increased awareness of the practical and ethical challenges of long term care in a nursing home, including complicated family dynamics, decreased quality of life, and the loss of independence. Students were able to identify positive coping strategies used by residents and caregivers to overcome these challenges. Negative coping strategies, denial and criticism of staff, were also identified.

Students reported an increase in patience and communication, verbal and nonverbal, skills. They were able to recognize the importance of communicating with respect and not condescension. In addition, students reported an increase in their ability to empathize with and offer compassion to elder adults. Lastly, students recognized the importance of effective team communication within interdisciplinary care teams.

## Conclusions

The “Ethics and Geriatric Care” undergraduate internship course at SLU has experienced significant success, specifically with regard to the goal of promoting awareness of geriatric medicine to pre-professional undergraduate students. This success is evident through the continued interest in the course shown through elective student enrollment. In addition, the post-course essay series and university conducted course analysis show high student satisfaction levels with the course. We hypothesize that the success of the course is related to three aspects of the course design: (1) students’ needs were integrated into the course design, (2) classroom instruction was enhanced by direct resident companionship and shadowing experiences and (3) the course integrates discussion of ethical dilemmas. The course is currently offered every semester and remains a popular elective for pre-professional students.

### Conflict of Interest

The authors declare that they have no conflict of interest.
